# Analysis of complete genomes of the rubella virus genotypes 1E and 2B which circulated in China, 2000–2013

**DOI:** 10.1038/srep39025

**Published:** 2016-12-13

**Authors:** Zhen Zhu, Min-hsin Chen, Emily Abernathy, Joseph Icenogle, Shujie Zhou, Changyin Wang, Chunfang Zhao, Yan Wang, Haiyun Chen, Yuan Si, Wenbo Xu

**Affiliations:** 1 WHO WPRO Regional Reference Measles/Rubella Laboratory and Key Laboratory of Medical Virology Ministry of Health, National Institute for Viral Disease Control and Prevention, Beijing, People’s Republic of China; 2 Measles, Mumps, Rubella, and Herpesviruses Laboratory Branch, National Center for Immunizations and Respiratory Diseases, Centers for Disease Control and Prevention, Atlanta, Georgia, USA; 3 Anhui Center for Disease Control and Prevention, Hefei, People’s Republic of China; 4 Shandong Center for Disease Control and Prevention, Jinan, People’s Republic of China; 5 Chongqing Center for Disease Control and Prevention, Chongqing, People’s Republic of China; 6 Liaoning Center for Disease Control and Prevention, Changchun, People’s Republic of China; 7 Hainan Center for Disease Control and Prevention, Haikou, People’s Republic of China; 8 Shaanxi Center for Disease Control and Prevention, Xi’an, People’s Republic of China

## Abstract

Rubella viruses of genotypes 1E and 2B are currently the most frequently detected wild-type viruses in the world. Genotype 1E viruses from China have been genetically distinct from genotype 1E viruses found elsewhere, while genotype 2B viruses found in China are not distinguishable from genotype 2B viruses from other areas. Genetic clusters of viruses of both genotypes were defined previously using sequences of the 739-nt genotyping window. Here we report phylogenic analysis using whole genomic sequences from seven genotype 1E and three genotype 2B viruses which were isolated in China between 2000 and 2013 and confirm the subgrouping of current circulating genotypes 1E and 2B viruses. In addition, the whole genomic characterization of Chinese rubella viruses was clarified. The results indicated that the Chinese rubella viruses were highly conserved at the genomic level, and no predicted amino acid variations were found at positions where functional domains of the proteins were identified. Therefore, it gives us the idea that the rubella control and elimination goal should be achieved if vaccine immunization coverage continues maintaining at the high level.

Rubella virus (RV), which causes low fever and mild rash, is the only member of the genus *Rubivirus* in the family *Togaviridae*. RV is one of the most potent teratogenic agents. Over 80% of congenital infections that occur in the first trimester of pregnancy lead to fetal death or congenital anomalies, also known as congenital rubella syndrome (CRS)^1^. Rubella vaccination is the most effective way to prevent rubella and CRS^2^. As of December 2014, 140 of the 194 WHO member states, including China, had introduced rubella vaccination as part of national immunization programs^3^.

RV has a single-stranded RNA genome of positive polarity of approximately 9762 nucleotides (nt). The genome encodes two open reading frames (ORFs). The 5′-proximal ORF in the RV genome encodes a non-structural protein (NSP), which has a molecular weight around 220-kDa and is processed into P150 and P90 by a virally encoded protease. The non-structural proteins are involved in RNA replication. The 3′-proximal ORF encodes viral structural proteins (SP), including the nucleocapsid protein (C) and two viral glycoproteins (E2 and E1)^1^. The genome also contains three untranslated regions (UTRs), including a 40-nt 5′UTR, a 118-nt intragenic region (IR) which can be variable in length and a 59-nt 3′UTR^1^. Amino acid sequence alignment between RV and other RNA viruses predicted several conserved domains in the NSP^4^. The P150 protein includes a methyl/guanylyltransferase domain (MT)^5^, two hypervariable regions (HVR)^6^, an X-domain (XD)^1^, and a non-structural protease (NP) domain^1^,^7^. P90 contains putative helicase (HEL) and RNA-dependent RNA polymerase (RdRp) domains^1^. Only the biochemical activities of NP and HEL have been confirmed experimentally^8^,^9^.

Phylogenic analysis of the nucleic acid sequences of a 739-nt region in E1 gene of wild-type RVs [corresponding to nts 8731–9469 in the RV reference strain (NC_001545.2)] showed two distinct groups, clade 1 and clade 2. The sequences in these two clades differ by ~8%. Within clade 1, one provisional (1a) and nine recognized genotypes (1B, 1C, 1D, 1E, 1F, 1G, 1H, 1I, 1J) have been designated; while three recognized genotypes have been designated in clade 2 (2A, 2B and 2C)^10^. Since 2012, genotypes 1E, 1G, 1J and 2B are the most frequently detected wild-type RVs and genotypes 1E and 2B have a global distribution^10^.

Currently there are approximately 50 whole genomic sequences of RV available in GenBank but approximately half are either vaccine or laboratory adapted strains. Whole genome sequences of four genotype 1E and seven genotype 2B viruses were previously determined^6^,^11^. In China, the previously endemic RV genotypes were likely 2A and 1F (before 2002) but both were replaced by genotype 1E^12^. Although genotype 1E is currently detected most frequently, an increasing number of genotype 2B viruses are now detected. Some of the genotype 2B viruses are believed to be imported, while others are likely due to endemic transmission^12^,^13^,^14^. Analysis based on 739-nt sequences, showed that viruses of genotype 1E from China were genetically distinct from viruses collected elsewhere and could be divided into two distinct clusters, Cluster A (1E-A) and Cluster B (1E-B)^13^. In addition, genotype 2B viruses found in China since 2011, although not distinct from those collected in other countries, were found to belong to two distinct lineages. The majority of 2B sequences were found in the previously designated Cluster C (2B-C)^13^, while three sequences found between 2001 and 2009 were located in a distinct 2B cluster which was not previously named. In this study, we performed genomic sequencing of genotypes 1E and 2B viruses isolated in China.

Virologic surveillance in China is currently by far the most complete of any country with 497 sequences reported from China compared to only 809 sequences reported from elsewhere in the world in the last 6 years^15^. This study extends previous work defining lineages within genotypes 1E and 2B using Chinese RV sequences from the 739 nt region of the E1 ORF. The lineages previously defined were confirmed, nt and amino acid (aa) variation across the entire RV genomes were analyzed, and possible functional variation in the viral proteins was considered. The results reported here add whole genome data to the as yet unexplained observation of changes in the predominant circulating RV(s) in rubella endemic countries^12^.

## Methods

### Viruses

The viruses were recovered from throat swabs from clinically confirmed rubella cases with written informed consent from all patients or legal guardians involved in this study. This study was approved by the second session of the Ethics Review Committee of the National Institute for Viral Disease Control and Prevention, Chinese Center for Disease Control and Prevention. And all methods were performed in accordance with the relevant guidelines and regulations.

Ten wild-type Chinese rubella virus isolates were chosen for whole genome sequencing because of the cluster to which their sequences belonged or because of their historical importance (Table 1). The viruses were isolated during routine virologic surveillance in Anhui, Shandong, Liaoning, Shaanxi, Chongqing, and Hainan provinces, covering the central, eastern and western regions of mainland China from 2000 to 2013. Viruses from throat swabs were propagated in Vero or Vero/SLAM cells as previously described^16^. Complete genome sequences of RV were determined using RNA from cells infected with RV, after two passages. One virus (RVi/Pullman.WA.USA/08[1E]) was isolated in the United States when a traveler from China became ill soon after arrival in the United States^6^. This virus, therefore, was also included for whole genome analysis (Table 1).

### Genome sequencing

Total RNA was extracted from infected cell cultures using the QIAamp Viral RNA Mini Kit (Qiagen; Valencia, CA, USA) according to the manufacturer’s instructions. Overlapping fragments (eight or nine for 1E and 2B viruses respectively), encompassing the entire genome were amplified by reverse transcription-polymerase chain reaction (RT-PCR) using the OneStep RT-PCR kit (Qiagen) according to the manufacturer’s protocol. All primers used in this study are listed in Supplemental Table 1. The PCR products obtained were gel purified using the QIAquick Gel extraction kit (Qiagen). The amplicons were bi-directionally sequenced using ABI Prism BigDye v3.1 terminator cycle sequencing kit and ABI PRISM 3130 genetic analyzer (Applied BioSystems; Foster City, CA, USA). The 5′and 3′ terminal sequences were determined by using the 5′/3′RACE kit (Roche Diagnostic, Mannheim, Germany) and oligo dT priming, respectively, as previously reported^6^. The primers were used as follows: 1R:.5′-GGC AAC CTC CCA TGA GAG TTC GGC C-3′; 2R: 5′-CAT CAG CTC GCA CAT CTG-3′; 3R: 5′-CGT CCT GTG GAG GCA CAG TG-3′.

### Phylogenetic and Bioinformatic analysis

Sequence editing was done using Sequencher version 4.0.5 (GeneCode, Ann Arbor, MI). The nucleotides and amino acid sequences were aligned using MEGA program (version 6.0) (Sudhir Kumar, Arizona State University, Tempe, Arizona, USA)^17^. Phylogenetic trees were constructed by the neighbor-joining method with the Kimura 2-parameters model and a maximum likelihood method with the Tamura-Nei model as implemented in MEGA program. The reliability of phylogenetic inference at each branch node was estimated by the bootstrap method with 1000 replications. Bootstrap values greater than 80% were considered statistically significant for grouping. The genetic distances (substitution/site) between clusters were calculated using p-distance in MEGA program^17^. This distance is the proportion (p) of nucleotide/amino acid sites at which to sequences being compared are different. It is obtained by dividing the number of nucleotide/amino acid differences by the total number of nucleotides compared. Evolutionary rates were estimated by a coalescent-based Bayesian method in BEAST (version 1.7.4)^18^. The Simplot program (version 3.5.1) was used for the recombination analysis^19^.

### Nucleotide sequence accession number

The 10 full-length RV genomic sequences determined in this study were deposited in GenBank (accession number: KT962862 to KT962871). The sequences were compared to the whole genomic sequences of 32 selected RV available in GenBank^6^,^11^ (Supplemental Table 2).

## Results

### Phylogenic analysis of Chinese genotypes 1E and 2B using complete genomic sequences

**Figure 1:**
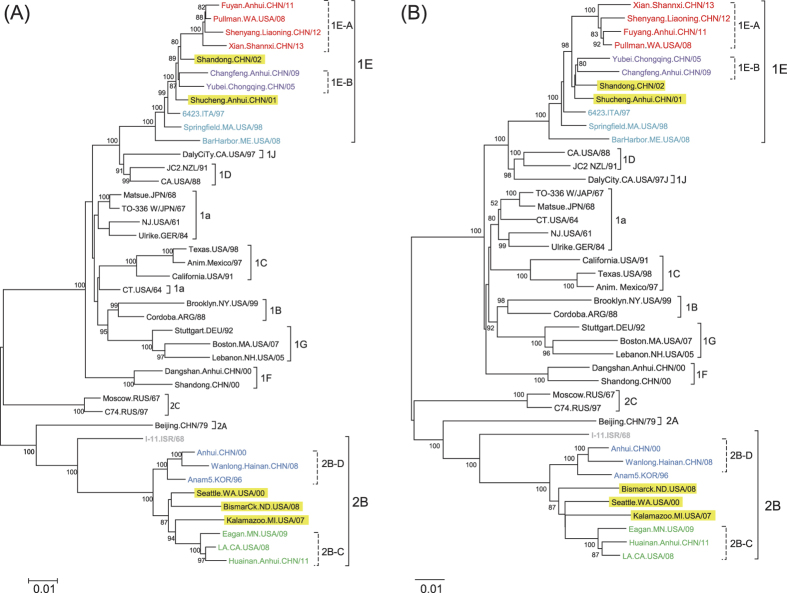
Phylogenetic tree of 42 RVs based on complete genomic sequences with maximum likelihood with Tamura-Nei model (**A**) or neighbor-joining method with Kimura 2-parameter model (**B**) by MEGA6. Clusters in 1E and 2B are color-coded and the outliers in 1E and 2B are highlighted. The percentage of replicate trees in which the associated genotypes/clusters were found by the bootstrap test (1000 replicates) are shown next to the branches. The distances were computed using the p-distance method and are in the units of the number of base differences per site. The tree is drawn to the scale shown at the bottom of the figure, with branch lengths measured in the number of substitutions per site. Each sequence is given an abbreviated name which is shown in Supplemental Table 1.

Whole genome sequencing was used to confirm the clustering of the Chinese genotypes 1E and 2B viruses that was based on the sequences of the 739-nt region of the E1 ORF. Selected viruses from each cluster found in China from 2000 to 2013 consisted of four representative viruses from 1E-A, two from 1E-B, one from 2B-C and two from a cluster of pre-2009 viruses designated as 2B-D (Table 1). Phylogenic analysis of the complete genomic sequences (Fig. 1A and B) by two models resulted in the same grouping as the results given by the analysis using 739-nt genotyping window, except that RVi/Shandong.CHN/02 was not grouped close to 1E-A by the neighbor-joining method (Fig. 1B). Three Chinese 1E-A viruses, RVi/Fuyang.Anhui.CHN/11, RVi/Shenyang.Liaoning.CHN/12, and RVi/Xian.Shannxi.CHN/13, were grouped together with RVi/Pullman.WA.USA/08, a documented export from China to the USA^6^. Using the maximum likelihood method with the Tamura-Nei model (Fig. 1A), the Chinese 1E sequences formed a genetic group distinct from non-Chinese sequences with bootstrap value of 99%, and the bootstrap value for subdividing 1E-A from 1E-B was 89%. Unlike genotype 1E, the Chinese genotype 2B sequences did not cluster as a distinct phylogenic group compared to genotype 2B viruses from other countries. With the exception of RVi/TelAviv.ISR/68 (DQ085338), the genotype 2B viruses formed two genetic lineages; subdivision of 2B-C from 2B-D was supported by a bootstrap value of 100 by both maximum likelihood and neighbor-joining methods (Fig. 1). Because RVi/TelAviv.ISR/68 is an outlier from the more recent genotype 2B RVs, this sequence was excluded from further analyses.

To determine whether the subdivisions of genotypes 1E and 2B were consistent throughout the entire genome, alignments from eighteen different regions of the genome were analyzed. These included the 5′ UTR, NSP, IR, SP and 3′UTR. Within NSP and SP, we also analyzed the subdivision by each protein (p150 and p90 in NSP and C, E2, E1 in SP) and domains. Except for the UTRs, HVRs and the MT, similar subdivisions were preserved among the other domains but the bootstrap values for subdivisions varied (data not shown). Shandong.CHN/02 was grouped with 1E-A viruses with 739-nt sequence; however, the grouping of this virus varied by different regions and by different methods (data not shown). Therefore, this virus was not considered as 1E-A or 1E-B. Similarly, RVi/Shucheng.Anhui.CHN/01, the earliest genotype 1E virus among the ten, was not grouped with 1E-A or 1E-B in most regions by maximal likelihood methods.

Subgrouping of genotype 2B was also supported by phylogeny using sequences of different regions/genes, except for the MT and the UTRs. Grouping of 2B-C and 2B-D are consistent along the entire genome by both methods. Two of the Chinese genotype 2B viruses (RVi/Anhui.CHN/00 and RVi/Wanlong.Hainan.CHN/08) consistently grouped together in 2B-D and in a distinct group from RVi/Huainan.Anhui.CHN/11, which was frequently grouped with two 2B-C viruses that were imported into USA (RVi/Eagen.MN.USA/09 and RVi/LA.CA.USA/08). The other three genotype 2B viruses, RVi/Seattle.WA.USA/00 and RVi/Kalamazoo.MI.USA/07 and RVi/Bismarck.ND.USA/08 (two of these were known importations into the USA from India and one was imported from an unknown source) were grouped with 2B-C using the whole genomic sequences by both Maximum Likelihood and Neighbor-joining methods. However, RVi/Bismarck.ND.USA/08, a virus imported from India, was no longer grouped with 2B-C when using the sequences of the 739-nt genotyping window by neighbor-joining method (Supplemental Figure 1B). The grouping of these three viruses can be different when using the sequences of different parts of the genome; therefore, these viruses were not considered as belonging to either the 2B-C or the 2B-D group.

### Characterization of the full-length RV genomic sequences

The genetic characteristics of the ten Chinese RV genomes determined in this work were consistent with others of the same genotype. All seven genotype 1E viruses were of the same length as the non-Chinese genotype 1E. As with all previously sequenced genotype 2B viruses, the three Chinese genotype 2B viruses contained a single nucleotide deletion at position 6422 in the IR^6^. All ten viruses consisted of a 40-nt 5′UTR, a 6351-nt NSP-ORF, a 120 or 119-nt IR, a 3192-nt SP-ORF, and a 59-nt 3′UTR.

The overall GC content of the new ten sequences was 69.7%, which was very close to 69.6% reported previously^6^. No predicted amino acid variations were found at positions where functional domains of the proteins were identified, such as the GDD motif in the RdRp domain^20^, the Zn binding domain (C1175, C1178, and C1227)^21^, the catalytic dyads of the viral protease (C1152 and H1273)^7^, the potential Ca^2+^-coordinating ligands (D1210 and D1217)^22^, the protease cleavage site (G1300)^7^, the retinoblastoma protein-binding motif (LPCAE; 1901–1905)^23^, N-linked glycosylation sites of E1 (N76, N177, N209)^24^,^25^, antigenic sites of E1 (aa 245–284)^26^, nor the hemagglutination inhibition and neutralization epitopes of E1 (aa 208–239)^26^.

Among the 42 whole genome sequences available nucleotide substitutions were detected most frequently at the third codon position (Supplemental Fig. 2A). Consistent with previous reports^6^, the highest nucleotide variability was found in HVR-II (nt 997–1031) in p150 (Supplemental Fig. 2B), in which 83% of positions contained variable nucleotides with an average pairwise distance (p-distance) of 0.147. The most conserved region was the MT domain with an average p-distance of less than 0.03. The two HVRs also exhibited high variability at the first and second codon positions (Supplemental Fig. 2A); as a result, they had the most variable predicted amino acid sequences. Approximately 69% and 82% of the positions were variable in HVR-I and HVR-II, respectively, while the MT domain had the most conserved amino acid sequences (Supplemental Fig. 2B). The E1 was the most conserved among the three SP proteins with average p-distances of 0.059 and 0.012 for nucleotides and amino acids, respectively; while the E2 ORF was the most variable with average p-distances of 0.068 and 0.035 for nucleotides and amino acids, respectively.

### Genetic relationship of genotypes 1E and 2B viruses

The overall mean distance among these 42 sequences is 0.06 (the average similarity of nucleotide is about 93.9%). Genotypes 1E and 2B are the most distant genotypes based on whole genome sequences. The two genotype 1E viruses, RVi/Fuyan.Anhui.CHN/11 and RVi/Pullman.WA.USA/08, are the closest with a p-distance of 0.006 while RVi/Xian.Shannxi.CHN/13 (a 1E-A virus) and genotype 2B RVi/Huainan.Anhui.CHN/11 (a 2B-C virus) are the most distant with a p-distance of 0.094. Genotype 1E and 2B remained the most distant genotypes based on the NSP or SP coding sequences. However, the genetic relationships can be different along the genome. For example, genotypes 1B and 2A are the most distantly related when comparing the sequences of the 5′UTR while genotypes 1F and 2A are the most distantly related when comparing the sequences of the 3′UTR.

**Figure 2:**
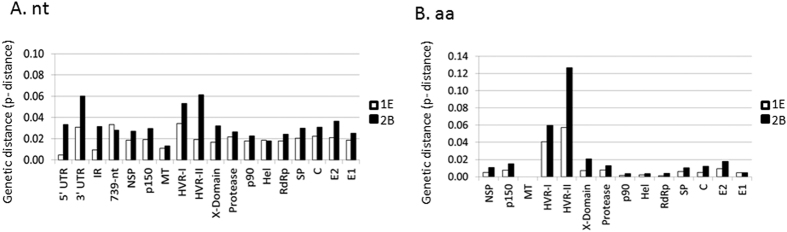
The estimated average divergence within genotypes 1E and 2B viruses based on nucleotide (nt) sequences (**A**) or predicted amino acid (aa) sequences (**B**). The evolutionary analyses are shown as the number of differences per site in each domain/gene and were obtained by averaging the number of differences for all sequence pairs in 1E and 2B.

In general, the genotype 2B is more diverse than the genotype 1E. The genetic distance within genotype 2B is 0.034; while it is 0.019 within 1E based on the whole genome sequences (Fig. 2). The 5′UTR had the most conserved nucleic acids (range from 0.000 to 0.025 in both genotypes 1E and in 2B) and the MT had the most conserved amino acid sequences (0.000 among viruses of both genotypes 1E and 2B). Indeed, among these 42 viruses, only one amino substitution at amino acid 131 (S in most clade 1 while G in 1J and clade 2) was found in these domains. The HVRs are the most variable coding regions of genotypes 1E and 2B: within genotype 1E, the average p-distance of HVR-I is 0.034 (range from 0.009 to 0.089) while surprisingly, the p-distance of HVR-II is 0.019, which is close to the overall distance using the whole genomic sequences. The p-distance of HVR-I and HVR-II within genotype 2B are 0.053 (range from 0.006 to 0.088) and 0.061 (range from 0.010 to 0.087), respectively. Among the three UTRs, 3′UTR is the most variable in most of the genotypes. In genotypes 1E and 2B, the average p-distances of 3′ UTR are 0.031 and 0.060, respectively (Fig. 2A). Although at the nucleic acid level, the p-distance of HVR-II is less than HVR-I in genotype 1E, at the amino acid level, HVR-II is the most variable in genotype 1E with p-distance of 0.057. In genotype 2B, HVR-II remains to be the most variable region at the amino acid level with p-distance of 0.127 (Fig. 2B).

Within the genotype 1E viruses, the non-Chinese genotype 1E contains more diverse sequences (p-distance = 0.019) than Chinese genotype 1E (p-distance = 0.017). Along the entire genome, the 3′ UTRs is one of the most variable regions with a p-distance of 0.045, which is only less than HVR-II (p-distance = 0.058) (Supplemental Fig. 3A). Within the Chinese genotype 1E viruses, the overall p-distances in 1E-A and 1E-B are 0.010 and 0.018, respectively, while the p-distance between the two outliers is 0.010. The two Chinese outliers are closer to the non-Chinese genotype 1E viruses (p distance = 0.017), compared to the distance to 1E-A or 1E-B (0.025 and 0.022 respectively (Supplemental Figure 3B).

The genotype 2B viruses found in China do not form a distinct phylogenic group from genotype 2B viruses found in other regions, nevertheless, two distinct phylogenic groups were noticed in post-1996 genotype 2B viruses based on complete genomic sequences. Creating subgroups within genotype 2B, especially 2B-C, is challenging as the sequences are more diverse than 1E and the number of viral sequences is limited. We sub-grouped genotype 2B viruses based on consistency by phylogeny, and as a result, three viruses that were initially sub-grouped with 2B-C using the 739-nt sequences are re-considered as outliers. The three genotype 2B outliers had greater genetic variability than the other two clusters (the p-distance using whole genome sequences of genotype 2B outliers, 2B-C and 2B-D are 0.028, 0.012 and 0.016, respectively). In general, the p-distances within and between genotype 2B groups are greater than the distances within and between genotype 1E groups. The three genotype 2B outliers show more diverse nucleic acid sequences along most of the genome except for the 3′ UTR, in which both genotype 2B clusters have p-distance of 0.068, the highest among other regions. (Supplemental Figure 4A). HVRs, especially HVR-II, remains the most variable coding region as the p-distance of amino acid sequences ranged from 0.059 (2B-C) to 0.098 (the three outliers) (Supplemental Figure 4A). Except for the 3′ UTR and HVRs, the p-distances between RVi/TelAviv.ISR/68 and 2B-C, 2B-D or genotype 2B outliers were similar in different regions of the genome (Supplemental Figure 4B).

### Cluster/genotype specific nucleic and amino acids in genotypes 1E and 2B

Thirteen clade specific amino acid variations were identified among 30 viruses^6^. Ten were preserved among the 42 viruses. Of these ten clade specific variations, 6 are in p150 and 4 are in SP. Six amino acid variations specific to genotype 1E were identified of which five were in p150 with three in the HVRII-protease domain, and one, A418T, was in E2. Five Chinese genotype 1E specific nucleotide substitutions were identified. None of these substitutions resulted in amino acid variation. Twelve 1E-A and one 1E-B specific nucleotide substitutions were found of which one, near the 5′ terminus of the genome (R1424T), resulted in an amino acid variation specific to 1E-A (Table 2).

Fifty-six nucleic acid substitutions and 16 amino acid variations specific to genotype 2B viruses were identified. Among the 16 amino acid variations, 11 were located in p150, two were in p90 (R/K1348M in helicase and M1739L in RdRp domains), and three were in the capsid protein. Nucleotide substitutions and/or amino acid variations specific to genotype 2B clusters were also identified (Table 3). The cluster-specific amino acid variations are either in p150 or in C/E2. None of these substitutions were located in the genotyping window.

### The evolutionary rate of RV and recombination

The evolutionary rates of global and Chinese genotype 1E viruses were estimated to be approximately 1.04 × 10^−3^ substitutions/site/year and 9.16 × 10^−4^ substitutions/site/year, respectively. The sequences of global genotype 2B viruses have an evolutionary rate of around 1.34 × 10^−3^ substitutions/site/year. No rate was calculated for UTRs and HVRII due to the short sequence lengths and rates given by HVR-I and X-domain were excluded because of low bootstrap values (Table 4).

Although genotype 1E-A and 2B-C viruses are co-circulating in China, no recombination event was found among these viruses using the similarity plot and boot scanning analysis in the Simplot program.

## Discussion

Genotypes 1E and 2B are currently the most frequently detected genotypes, with a worldwide distribution that includes China^10^. A genotype 1E sequence was first reported in France in 1995^27^ and was predicted to have emerged around the early 1990’s^28^. Since then, genotype 1E viruses have been found in Europe, Southeast Asia, and northern Africa with sporadic cases or outbreaks occurring in other regions such as the Americas^10^,^29^. In China, genotype 1E was first reported in 2001^12^ and it has been the dominant RV genotype since then. Phylogenetic analysis of genotype 1E viruses collected from 2001 to 2012 based on the 739-nt genotyping window showed that the Chinese genotype 1E viruses appeared to be a distinct lineage from the genotype 1E viruses found elsewhere^12^,^13^,^28^. This divergence has become more prominent in recent Chinese genotype 1E viruses as the subgroup (1E-A) is now distinct from earlier sequences.

In this report, we confirmed and refined the subdivision of Chinese genotype 1E viruses using complete genomic sequences. To put the conclusions from the present work in context, it is necessary to include conclusions previously derived from analysis of the large number of 739-nt sequences available^13^. The two older genotype 1E viruses collected in China in 2001 and 2002 were close to two of the non-Chinese genotype 1E sequences isolated in late 1990’s, indicating that the 2001 and 2002 viruses may have been introduced from outside China soon after genotype 1E viruses were first found. The earliest 1E-A sequence was found in China in 2004. Two hundred twenty seven genotype 1E sequences were reported in China during 2001–2009, of which 170 were 1E-A sequences. In 2010, rubella virologic surveillance was greatly enhanced in China and detection of subgroup 1E-A increased. Viruses in clusters 1E-A and 1E-B co-circulated during 2004 and 2009 and 1E-A viruses appear to have replaced 1E-B viruses in China around 2010. Viruses in cluster 1E-B, which have not been detected since 2009, were more diverse than 1E-A^13^.

Genotype 2B likely emerged in the early 1960’s^13^ and has recently become the most widely distributed global genotype. Approximately 300 nucleotide variations were found between RVi/TelAviv.ISR/68, the earliest documented genotype 2B virus, and the nine post-1996 genotype 2B whole genome sequences. This is less than the estimated change (~520 substitutions) calculated by the evolutionary rate of 1.34 × 10^−3^ substitution/site/year. Consistent with a previous study^6^, all genotype 2B viruses preserved a single nucleotide deletion relative to the RV reference sequence (CT.USA/64) at nt 6422 in the IR. The same deletion was present in RVi/TelAviv.ISR/68, but not in other Clade 2 genotypes. However, other sequences in the IR of RVi/TelAviv.ISR/68 are not genotype 2B specific. Therefore phylogenic analysis using the IR doesn’t map RVi/TelAviv.ISR/68 with genotype 2B. Although the deletion at nt 6422 suggests a relationship to post-1996 genotype 2B viruses, there are insufficient sequences between 1968 and 1996 to establish the precise relationship between RVi/TelAviv.ISR/68 and current clade 2 viruses.

Unlike the genetically distinct Chinese genotype 1E viruses, the Chinese genotype 2B viruses grouped into two clusters containing genotype 2B viruses from other countries. RVi/Huainan.Anhui.CHN/11 was always grouped with RVi/LA.CA.USA/08 (an importation from India into the USA) in 2B-C, while RVi/Anhui.CHN/00 and RVi/Wanlong.Hainan.CHN/08 were always grouped together in 2B-D along the entire genome except the UTRs and HVR-II. 2B-C viruses are the current circulating cluster in China. The three genotype 2B outliers may represent other genotype 2B clusters. Again, more sequences from other countries are needed to further characterize the diversity in genotype 2B viruses. Currently, the viruses of genotype 1E from China offer the most complete data set available to study RV evolution. Nevertheless, useful data from genomic sequences of both genotypes 1E and 2B viruses were obtained in this study.

The ten viruses selected for complete genomic sequencing were collected over a period of 13 years. No predicted amino acid variations specific to all Chinese genotype 1E viruses were found. However, a cysteine residue at amino acid 462 in p150 was preserved in all 1E-A viruses, but can be glycine, serine or valine in other genotypes and 1E-B viruses. No amino acid variations or nucleotide substitutions specific to all Chinese genotype 2B viruses were found. Sixteen unique amino acid residues were preserved in the nine post-1996 genotype 2B viruses and 10 of them were within the N-terminal 800 amino acids of NSP. None of these changes occurred in E1, the major immunogenic protein, nor were any associated with known functional domains in the other proteins. RV exhibited remarkable conservation of amino acid residues in all known functional domains^30^. This is consistent with our observation that most nucleotide substitutions are transitional and occur at the 3^rd^ codon positions.

The genetic variability of RNA viruses allows viruses to adapt to new environments and escape host immune responses. Such variability can result from errors introduced by replication proteins or recombination, as is seen in human enterovirus^31^. No clear evidence of recombination was found among the ten RVs analyzed here. This is consistent with a previous report^6^, indicating that RNA recombination is probably not a crucial force to drive RV evolution. We estimated that the evolutionary rates of genotypes 1E and 2B were almost the same and would be approximately 1 × 10^−3^ substitutions/site/year. However, different regions of the genome appeared to evolve at different rates. These rates are faster than the average for alphaviruses (1–8 × 10^−4^ substitutions/site/year)^32^ and measles virus (0.65 × 10^−3^ substitutions/site/year based on the the H gene)^33^ but slower than polio virus (synonymous substitutions rate ~1 × 10^−2^ substitutions/site/year)^34^. RV is known to have a high GC content and slow replication in tissue culture. It is not clear whether such properties are related to the relatively low level of variability and evolutionary rate.

Here we report phylogenic analysis using whole genomic sequences from China and confirm the subgrouping of current circulating genotypes 1E and 2B viruses. To achieve global rubella elimination by 2020, it is important to enhance virologic surveillance of endemic strains in China. In this study, the whole genomic characterization of Chinese rubella viruses was clarified. The results indicated that the Chinese rubella viruses were highly conserved at the genomic level, and no predicted amino acid variations were found at positions where functional domains of the proteins were identified after long-term circulation. Therefore, it gives us the idea that the rubella control and elimination goal should be achieved if vaccine immunization coverage continues maintaining at the high level.

## Additional Information

**How to cite this article**: Zhu, Z. *et al*. Analysis of complete genomes of the rubella virus genotypes 1E and 2B which circulated in China, 2000–2013. *Sci. Rep.* **6**, 39025; doi: 10.1038/srep39025 (2016).

**Publisher’s note:** Springer Nature remains neutral with regard to jurisdictional claims in published maps and institutional affiliations.

## Supplementary Material

Supplementary Tables and Figures

## Figures and Tables

**Table 1:** Endemic and exported Chinese rubella viruses used in this study.

Strain	Genotype/cluster	Isolation province and year	Length of whole genome sequences	GenBank Accession No.	Note
RVi/Shucheng.Anhui.CHN/25.01/2[1E]	1E outlier	Anhui, 2001	9762nt	KT962864	The earliest Chinese 1E strain
RVi/Shandong.CHN/0.02[1E]	1E outlier	Shandong, 2002	9762nt	KT962870	WHO reference strain
RVi/Fuyang.Anhui.CHN/52.11/3[1E]	1E-A	Anhui, 2011	9762nt	KT962866	
RVi/Shenyang.Liaoning.CHN/11.12/1[1E]	1E-A	Liaoning, 2012	9762nt	KT962869	
RVi/Xian.Shannxi.CHN/13.13/1[1E]	1E-A	Shaanxi, 2013	9762nt	KT962871	
RVi/Pullman.WA.USA/08[1E]	1E-A	NA, 2008	9762nt	GU353078.1	Imported to USA*
RVi/Yubei.Chongqing.CHN/25.05/1[1E]	1E-B	Chongqing, 2005	9762nt	KT962867	
RVi/Changfeng.Anhui.CHN/15.09[1E]	1E-B	Anhui, 2009	9762nt	KT962863	
RVi/Anhui.CHN/0.00/2[2B]	2B-D	Anhui, 2000	9761nt	KT962862	WHO reference strain
RVi/Wanlong.Hainan.CHN/2.08/6[2B]	2B-D	Hainan,2008	9761nt	KT962868	
RVi/Huainan.Anhui.CHN/41.11 [2B]	2B-C	Anhui, 2011	9761nt	KT962865	

^*^Note: From rubella case exported to the USA from China, verified by epidemiological data.

**Table 2:** Nucleotides (nt) and amino acids (aa) preserved in genotype 1E subgroups among 42 RV genomes.

			1E-Chinese		1E-A		1E-B	
			nt	aa	nt	aa	nt	aa
**NSP**	**p150**	**Unknown (440–2119)**	C1021T		**R1424T**	**(G/S/V)462C**		
		**HVR-I (2120–2440)**			C2413T			
		**X-domain (2492–2998)**			C2893T		T2796C	
		**HVR-II (3029–3133)**	G3709A		**M3538T**			
		**& Protease (3035–3973)**			C3691T			
					**M3712G**			
	**p90**	**Hel (4043–4798)**			**Y4753G**			
		**RdRp (4826–6388)**	**W5521G**		**R4900C**			
			C5902T		R5236C			
					**R5428T**			
					**C5431T**			
**SP**	**C (6512–7411)**				**Y7303A**			
	**E1 (8258–9703)**		**Y8623A**					

Bold values indicates that heterogeneous variability was also present in other genotypes at the indicated position. IUPAC code was used for nt variation.

**Table 3:**
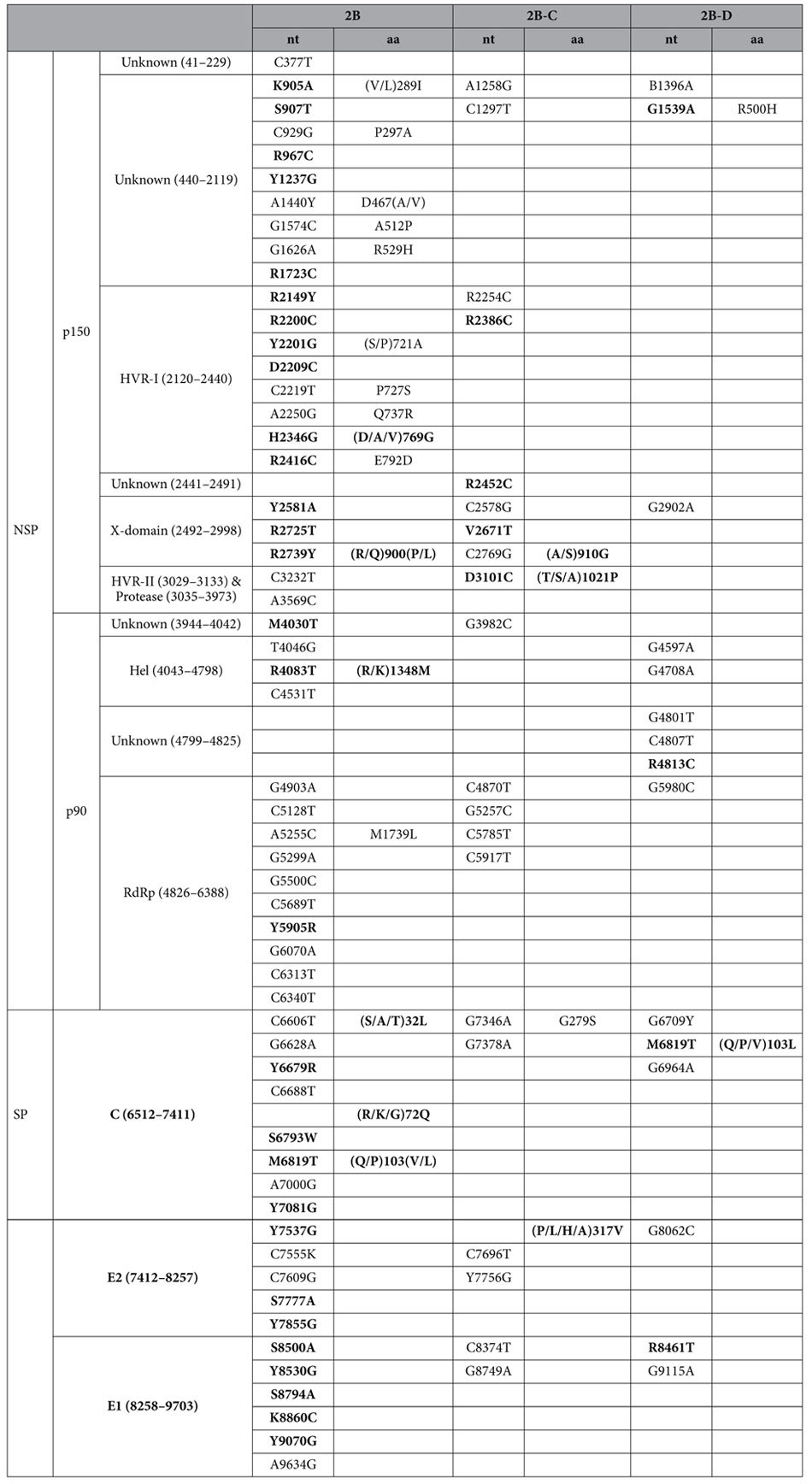
Nucleotides (nt) and amino acids (aa) preserved in genotype 2B subgroups among 42 RV genomes.

Bold vaules indicates that heterogeneous variability was also present in other genotypes at the indicated position. IUPAC code was used for nt variation.

**Table 4:** Evolution rate of 1E and 2B viruses in different domains^1^.

Region	Genotype 1E	Genotype 2B
Whole genome	1.04 × 10^−3^(95%HPD:5.02 × 10^−4^–1.59 × 10^−3^)	1.34 × 10^−3^(95%HPD:2.34 × 10^−4^–1.56 × 10^−3^)
5′UTR	na	na
NSP	1.14 × 10^−3^(95%HPD:5.38 × 10^−4^–1.74 × 10^−3^)	1.36 × 10^−3^(95%HPD:4.97 × 10^−4^–2.13 × 10^–3^)
p150	1.10 × 10^−3^(95%HPD:4.66 × 10^−4^–1.73 × 10^−3^)	1.64 × 10^−3^(95%HPD:7.31 × 10^−4^–2.51 × 10^−3^)
MT	2.34 × 10^-2^(95%HPD:2.11 × 10^-7^–9.50 × 10^−2^)	2.55 × 10^−3^(95%HPD:7.58 × 10^−7^–8.08 × 10^−3^)
HVR-I(low BT)	8.97 × 10^−3^(95%HPD:5.56 × 10^−4^–2.34 × 10^−2^)	3.02 × 10^−3^(95%HPD:5.30 × 10^−4^–5.69 × 10^−3^)
HVR-II(low BT)	na	na
X-Domain (low BT)	2.11 × 10^−3^(95%HPD:4.92 × 10^−4^–4.18 × 10^−3^)	3.66 × 10^−3^(95%HPD:3.60 × 10^−4^–8.88 × 10^−3^)
Pro	1.35 × 10^−3^(95%HPD:4.63 × 10^−4^–2.15 × 10^−3^)	2.08 × 10^−3^(95%HPD:4.92 × 10^−4^–3.48 × 10^−3^)
NP	1.49 × 10^−3^(95%HPD:4.67 × 10^−4^–2.42 × 10^−3^)	1.86 × 10^−3^(95%HPD:7.15 × 10^−4^–3.02 × 10^−3^)
p90	1.27 × 10^−3^(95%HPD:5.92 × 10^−4^–1.97 × 10^−3^)	1.02 × 10^−3^(95%HPD:2.81 × 10^−4^–1.76 ×10^−3^)
Hel	1.63 × 10^−3^(95%HPD:3.25 × 10^−4^–3.05 × 10^−3^)	1.38 × 10^−3^(95%HPD:1.44 × 10^−4^–2.66 × 10^−3^)
RdRp	2.66 × 10^−3^(95%HPD:2.55 × 10^−4^–6.65 × 10^−3^)	1.11 × 10^−3^(95%HPD:2.99 × 10^−4^–1.90 × 10^−3^)
IR	na	na
SP	1.16 × 10^−3^(95%HPD:5.28 × 10^−4^–1.74 × 10^−3^)	1.44 × 10^−3^(95%HPD:5.96 × 10^−4^–2.20 × 10^−3^)
C	1.79 × 10^−3^(95%HPD:6.52 × 10^−4^–3.09 × 10^−3^)	1.75 × 10^−3^(95%HPD:6.21 × 10^−4^–2.83 × 10^−3^)
E2	1.56 × 10^−3^(95%HPD:6.65 × 10^−4^–2.51 × 10^−3^)	1.96 × 10^−3^(95%HPD:6.88 × 10^−4^–3.15 × 10^−3^)
E1	1.15 × 10^−3^(95%HPD:3.86 × 10^−4^–1.81 × 10^−3^)	1.34 × 10^−3^(95%HPD:5.54 × 10^−4^–2.19 × 10^−3^)
739-nt	1.82 × 10^−3^(95%HPD:3.75 × 10^−4^–3.24 × 10^−3^)	1.76 × 10^−3^(95%HPD:6.92 ×10^−4^–2.85 × 10^−3^)
3′UTR	na	na

^1^including 11 genotype 1E viruses and 10 genotype 2B viruses (see Table 1 and Supplemental Table 2); na: not available.
