# Supplementary material for: Analysis of complete genomes of the rubella virus genotypes 1E and 2B which circulated in China, 2000–2013
Source: Sci Rep. 2016 Dec 13;6:39025. doi: 10.1038/srep39025 (PMC5154293; doi:10.1038/srep39025)

Analysis of complete genomes of the rubella virus genotypes 1E and 2B which circulated in China, 2000-2013

Zhen Zhu[1*], Min-hsin Chen[2*], Emily Abernathy[2], Joseph Icenogle[2], Shujie Zhou[3], Changyin Wang[4], Chunfang Zhao[5], Yan Wang[6], Haiyun Chen[7], Yuan Si[8], and Wenbo Xu[1§]

Supplemental Table 1. Primers used for amplification and sequencing of genotype 1E and 2B RVs

Genotype	Position*	Sequence (5’-3’ orientation)	Forward/reverse
1E	1-22	CAATGGGAGCTATCGGACCTCG	Forward
	1607-1624	CTCGGCGAACAGCCACGG	Reverse
	1367-1386	GAGGAGTGGGAACAGGACGC	Forward
	3149-3168	GTGTACCGGCAGAGTTCGCA	Reverse
	2993-3013	GCGGGGCTCGCTGCCAGGCGC	Forward
	4613-4632	GTGTATGCGCGTATGCCAGC	Reverse
	4346-4365	GCGGAGGTGATCTGCGTCGG	Forward
	5646-5666	CGACCTCGATGGCATTGGTGG	Reverse
	5246-5263	GAGTGGCGCCTGACGTAC	Forward
	6543-6561	TCGAGGGCCTTCTGGAGGT	Reverse
	6366-6383	GCGCCAACCTCCACGACG	Forward
	7479-7499	GGTGGTGATGGCCGTAGTGCT	Reverse
	7217-7236	GGCACGCGCTGGCACCGACT	Forward
	8426-8445	CCCAAGCCCTCGCACGAGAC	Reverse
	8251-8271	CTATGGCGAGGAGGCTTTCAC	Forward
	9737-9762	CTATACAGCAACAGGTGCGGGAATCT	Reverse
2B	1-22	CAATGGGAGCTATCGGACCTCG	Forward
	1607-1624	CTCGGCGAACAGCCACGG	Reverse
	1364-1383	ATGGAGGAGTGGGAACAGGA	Forward
	2706-2725	AGCGACCGCGTGGATGATGT	Reverse
	2587-2604	CGTCTGCGGTGCCATCTT	Forward
	3808-3827	CGCGCACTTCGAGCACCACG	Reverse
	2993-3013	GCGGGGCTCGCTGCCAGGCGC	Forward
	4613-4632	GTGTATGCGCGTATGCCAGC	Reverse
	4346-4365	GCGGAGGTGATCTGCGTCGG	Forward
	5646-5666	CGACCTCGATGGCATTGGTGG	Reverse

5246-5263	GAGTGGCGCCTGACGTAC	Forward
6542-6560	TCGAGGGCCTTCTGGAGGT	Reverse
6366-6383	GCGCCAACCTCCACGACG	Forward
7478-7498	GGTGGTGATGGCCGTAGTGCT	Reverse
7325-7345	ATCCTTGGCGCATCCGCTTC	Forward
8389-8408	CAGATGCAGGCTCCGGTGGC	Reverse
8250-8270	CTATGGCGAGGAGGCTTTCAC	Forward
9736-9761	CTATACAGCAACAGGTGCGGGAATCT	Reverse

*Genotype 1E primers were designed based on the 1E sequence 6423_ITALY_1997 (GenBank accession number DQ085343); genotype 2B primers were designed based on the 2B sequence Anam5_Korea_1996 (GenBank accession number DQ085342).

Supplemental Table 2. 32 selected RV sequences used for phylogenetic analysis

Virus	Abbreviation	Genotype	Strain	GenBank Accession number
Rvi/NewJersey.USA/61	NJ.USA/61	1a	M33; lab strain	JN635281
RV/Connecticut.USA/0.64	CT.USA/64	1a	F-therien; lab strain and NCBI reference sequence	NC001545
RVi/Leipzig.DEU/84	Ulrike.GER/84	1a	Wild-type	AF435865
RVi/Toyama.JPN/0.67	TO-336/JPN/67	1a	Wild-type	AB047330
RVi/Matsue.JPN/68	Matsue.JPN/68	1a	Vaccine	AB222609
RVi/Brooklyn.NY.USA/15.99	Brooklyn.NY.USA/99	1B	Wild-type	JN635285
RVi/Cordoba.ARG/88	Cordoba.ARG/88	1B	Wild-type	DQ085339
RVi/California.USA/91	California.USA/91	1C	Wild-type	JN635283
RVi/Texas.USA/34.98	Texas.USA/98	1C	Wild-type	JN635284
RVi/Mexico/97	Anim.Mexico/97	1C	Wild-type	DQ085341
RVi/California.USA/88	CA.USA/88	1D	Wild-type	JN635285
RVs/JC2.NZL/91	JC2.NZL/91	1D	Wild-type	DQ388281
RV/BarHarbor.ME.USA/43.08	BarHarbor.ME.USA/08	1E	Wild-type	JN635286
RVi/Springfield.MA.USA/49.98	Springfield.MA.USA/98	1E	Wild-type	JN635287
RVi/Pullman.WA.USA/30.08	Pullman.WA.USA/08	1E	Wild-type	JN635288
RVi/6423.ITA/1997	6423/ITA97	1E	Wild-type	DQ085343
RVi/Shandong.CHN/15.00/1	Shandong.CHN/00	1F	Wild-type	JQ624625
RVi/Dangshan.Anhui.CHN/39.00/3	Dangshan.Anhui.CHN/00	1F	Wild-type	JQ624624
RVi/Boston.MA.USA/13.07	Boston.MA.USA/07	1G	Wild-type	JN635289
RVi/Lebanon.NH.USA/3.05	Lebanon.NH.USA/05	1G	Wild-type	JN635290
Stuttgart.DEU/92	Stuttgart.DEU/92	1G	Wild-type	DQ388280
RVi/DalyCity.CA.USA/.97	DalyCity.CA.USA/97	1J	Wild-type	JN635291
RVi/Beijing.CHN/79	BR1-CHN/79	2A	Wild-type	AY258322
RVi/TelAviv.ISR/68	I-11.ISR/68	2B	Wild-type	DQ085338
RVi/Kalamazoo.MI.USA/4.07-1	Kalamazoo.MI.USA/07	2B	Wild-type	JN635292
RVi/Seattle.WA.USA/16.00	Seattle.WA.USA/00	2B	Wild-type	JN635293
RV/LA.CA.USA/45.08CRS	LA.CA.USA/08	2B	Wild-type	JN635294
RVi/Eagan.MN.USA/13.09	Eagan.MN.USA/09	2B	Wild-type	JN635295
RVi/Bismarck.ND.USA/23.08	BismarCk.ND.USA/08	2B	Wild-type	JN635296
RVi/Anam5.KOR/1996	Anam5.KOR/96	2B	Wild-type	DQ085342
RVi/Moscow.RUS/1967	Moscow.RUS/67	2C	Unknown	DQ388279
RVi/C74.RUS/1997	C74.RUS/97	2C	Unknown	DQ085340

Supplemental Figure 1. Phylogenetic analysis of 42 Rubella viruses using the sequences of the 739-nt genotyping window using the Maximum Likelihood method based on the Tamura-Nei model (A) or the Neighbor-Joining method (B). The tree is drawn to scale at the bottom of the figure, with branch lengths measured in the number of substitutions per site.

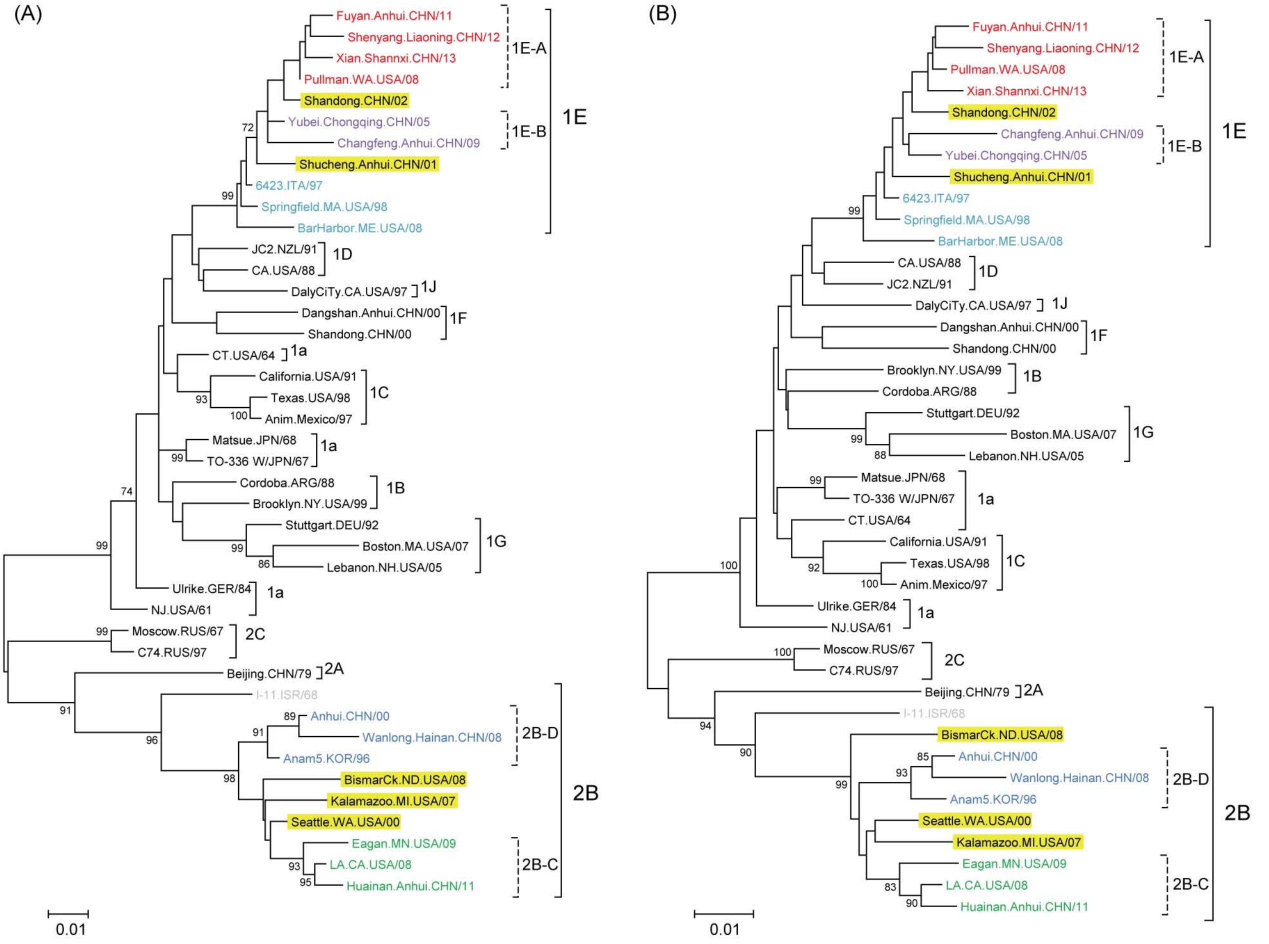
(A)
Fuyan.Anhui.CHN/11
Shenyang.Liaoning.CHN/12
Xian.Shannxi.CHN/13
Pullman.WA.USA/08
Shandong.CHN/02
Yubei.Chongqing.CHN/05
Changfeng.Anhui.CHN/09
Shucheng.Anhui.CHN/01
6423.ITA/97
Springfield.MA.USA/98
BarHarbor.ME.USA/08
1E-A
1E-B
1E
JC2.NZL/91
CA.USA/88
1D
DalyCiTy.CA.USA/97
1J
Dangshan.Anhui.CHN/00
Shandong.CHN/00
1F
CT.USA/64
1a
California.USA/91
Texas.USA/98
Anim.Mexico/97
1C
Matsue.JPN/68
TO-336 W/JPN/67
1a
Cordoba.ARG/88
Brooklyn.NY.USA/99
1B
Stuttgart.DEU/92
Boston.MA.USA/07
Lebanon.NH.USA/05
1G
Ulrike.GER/84
NJ.USA/61
1a
Moscow.RUS/67
C74.RUS/97
2C
Beijing.CHN/79
2A
I-11.ISR/68
Anhui.CHN/00
Wanlong.Hainan.CHN/08
Anam5.KOR/96
2B-D
BismarCk.ND.USA/08
Kalamazoo.MI.USA/07
Seattle.WA.USA/00
Eagan.MN.USA/09
LA.CA.USA/08
Huainan.Anhui.CHN/11
2B-C
2B
0.01
(B)
Fuyan.Anhui.CHN/11
Shenyang.Liaoning.CHN/12
Pullman.WA.USA/08
Xian.Shannxi.CHN/13
Shandong.CHN/02
Changfeng.Anhui.CHN/09
Yubei.Chongqing.CHN/05
Shucheng.Anhui.CHN/01
6423.ITA/97
Springfield.MA.USA/98
BarHarbor.ME.USA/08
1E-A
1E-B
1E
CA.USA/88
JC2.NZL/91
1D
DalyCiTy.CA.USA/97
1J
Dangshan.Anhui.CHN/00
Shandong.CHN/00
1F
Brooklyn.NY.USA/99
Cordoba.ARG/88
1B
Stuttgart.DEU/92
Boston.MA.USA/07
Lebanon.NH.USA/05
1G
Matsue.JPN/68
TO-336 W/JPN/67
CT.USA/64
1a
California.USA/91
Texas.USA/98
Anim.Mexico/97
1C
Ulrike.GER/84
NJ.USA/61
1a
Moscow.RUS/67
C74.RUS/97
2C
Beijing.CHN/79
2A
I-11.ISR/68
BismarCk.ND.USA/08
Anhui.CHN/00
Wanlong.Hainan.CHN/08
Anam5.KOR/96
2B-D
Seattle.WA.USA/00
Kalamazoo.MI.USA/07
Eagan.MN.USA/09
LA.CA.USA/08
Huainan.Anhui.CHN/11
2B-C
2B
0.01

Supplemental Figure 2. The average genetic distance of all 42 rubella viruses in different regions. A. Nucleotide transitions (Ts) and transversions (Tv) at the 1st, 2nd and 3rd codon position; B. The genetic distance of nucleotide (nt) and amino acid (aa).

A. Variability at coding positions in different domains

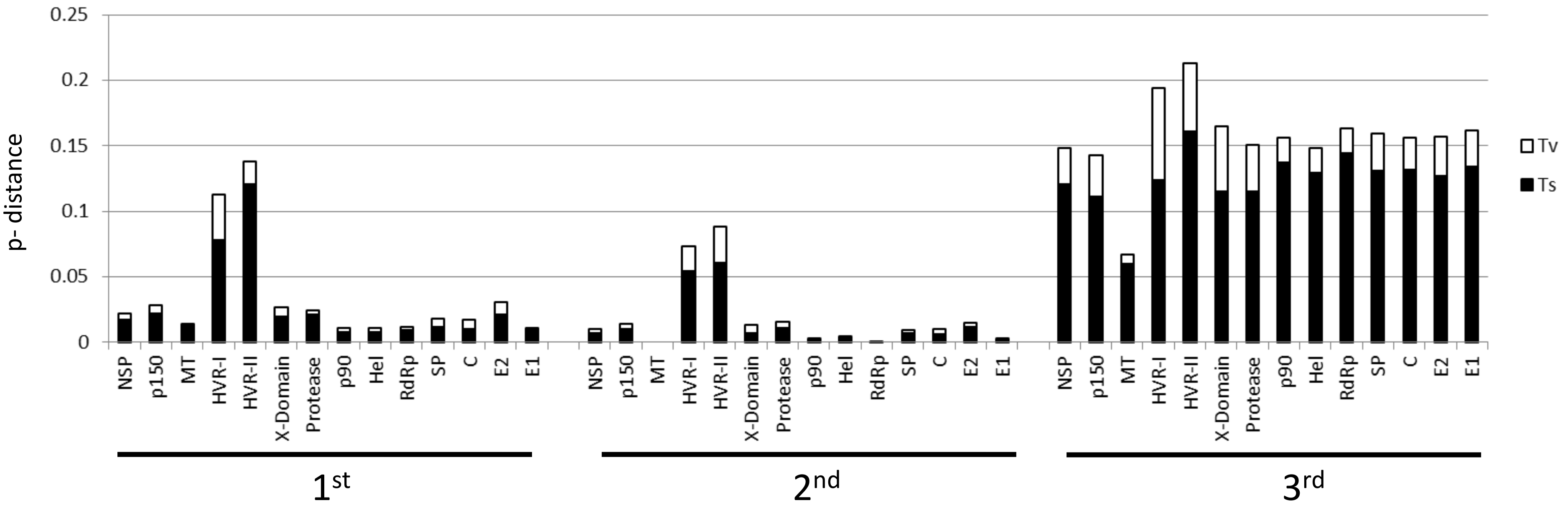

B. Average genetic distance of all 42 viruses in different domains

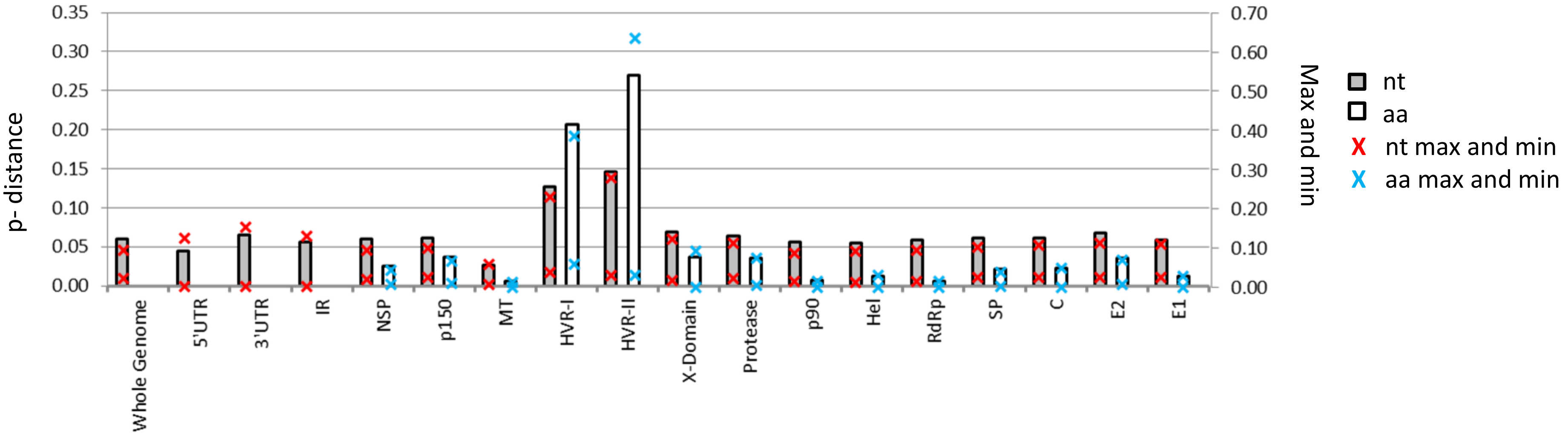

Supplemental Figure 3. The genetic variability within (A) and among (B and C) different clusters of genotype 1E rubella viruses. The methods for estimating genetic divergence within or between clusters pairwise and graphing are as described in Figure 2.

A. Genetic variability within 1E clusters

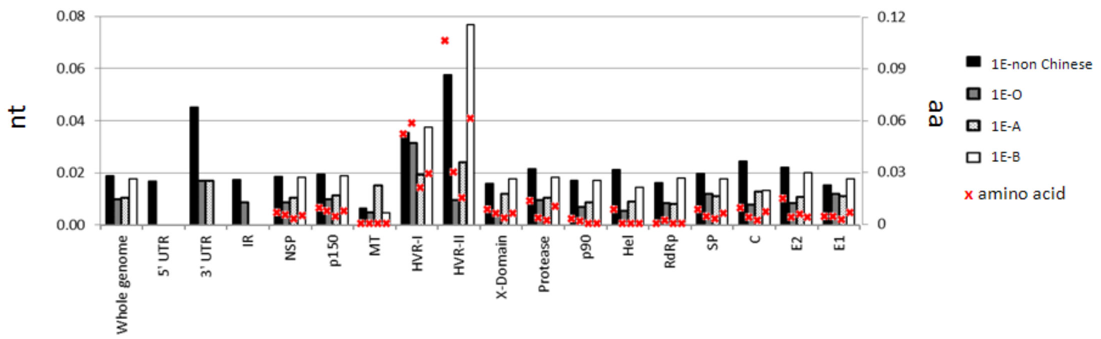

B. Genetic relationship between non-Chinese 1E and Chinese 1E groups

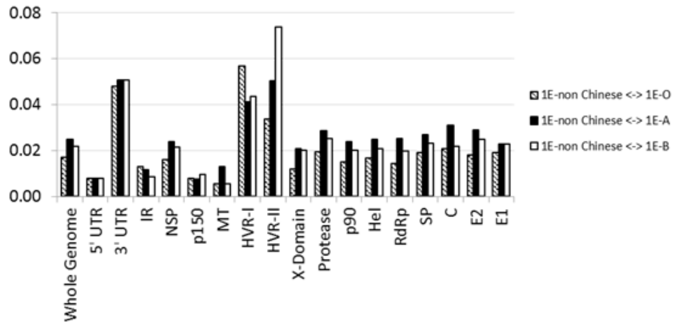

C. Genetic relationship between Chinese 1E groups

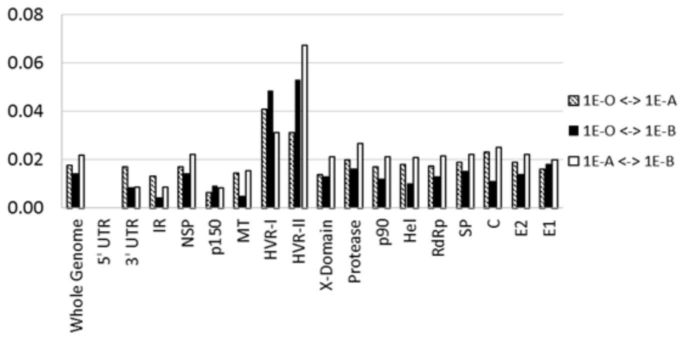

Supplemental Figure 4. The genetic variability within (A) and between (B and C) different clusters of genotype 2B rubella viruses. The methods for estimating genetic divergence within or between clusters pairwise and graphing are as described in Figure 2.

A. Genetic variability within 2B clusters

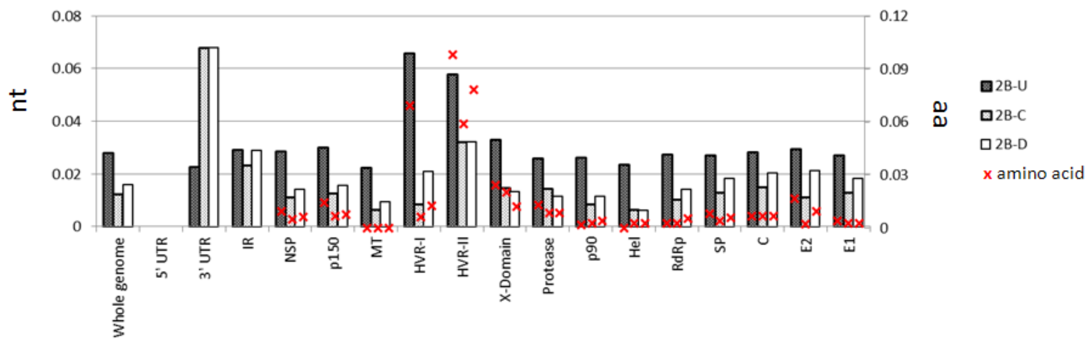

B. Genetic relationship between strain I-11 and 2B clusters

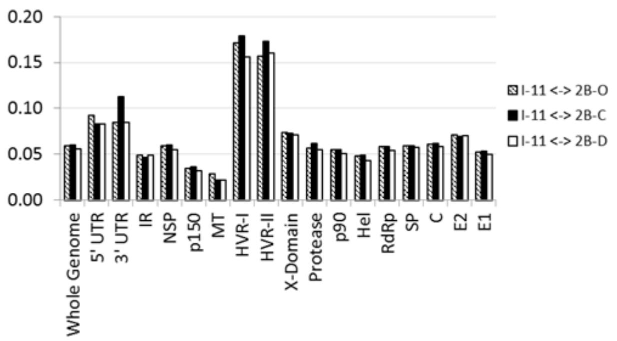

C. Genetic relationship among 2B clusters

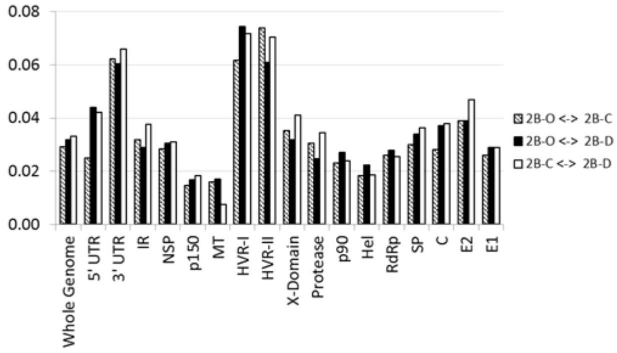

Supplement: Supplementary Tables and Figures [file srep39025-s1.pdf]
